# Editorial: Health education and type 2 diabetes

**DOI:** 10.3389/fnut.2023.1325517

**Published:** 2023-11-17

**Authors:** Luciana Neri Nobre, Edson da Silva

**Affiliations:** Program in Sciences of Nutrition, Nutrition Department, Universidade Federal dos Vales do Jequitinhonha e Mucuri, Diamantina, Brazil

**Keywords:** health education, diabetes education, type 2 diabetes, preventive measures, self-management skills

Diabetes mellitus (DM) is a chronic condition on the rise globally. The International Diabetes Federation (IDF) estimated that around 537 million adults were affected by diabetes in 2021 worldwide and that 3 in 4 adults with diabetes live in low- and middle-income countries. Furthermore, it was estimated that 541 million people had impaired glucose tolerance. These estimates included all types of diabetes: type 1 (DM1), type 2 (DM2), and Gestational Diabetes Mellitus (1). The increasing prevalence of DM is stimulated by a complex interaction of socioeconomic, demographic, environmental, and genetic factors. The continuous increase is mainly due to the increase in type 2 diabetes (DM2) and related risk factors, which include increasing urbanization and changing lifestyle habits, with a growing increase in obesity, food consumption of unhealthy, energy-rich, ultra-processed foods, and sedentary lifestyles (1).

DM2 is the most common form, responsible for more than 90% of all types of diabetes worldwide, and which causes high rates of morbidity and early mortality. This chronic condition can occur at any age but is more common in adults and the elderly (1, 2) and is a very costly disease for individuals and society globally (1, 3).

In addition to the high costs, DM can trigger several complications for people living with diabetes, and they may be associated with the low problem-solving capacity of health systems and the low awareness of professionals regarding prevention, factors that imply ignorance of the condition, poor adherence to treatment, and deficiency in self-management of care (4).

However, it is possible to reduce the impact of diabetes with preventive measures for DM2, early diagnosis, adequate care for all types of diabetes, and especially with health education. These measures can help people live better with DM, avoiding or delaying its complications (5).

It is noteworthy that diabetes education is related to the continuous and collaborative process of developing specific skills and incorporating the tools necessary to achieve the goals established at each stage of DM treatment (6). In this sense, diabetes education is considered part of the treatment itself, and for this reason, it must be included in all levels of care for people with diabetes so that they can be active in their treatment. It can even be said that diabetes education is one of the pillars of treating this condition. It intervenes directly in improving the quality of life of these people, giving them the right to decide the most appropriate strategies to promote, maintain, and recover health (7).

Health education can be defined as a set of pedagogical, participatory practices, that encompasses knowledge that involves different fields of activity and that empowers individuals and communities to develop their capabilities as a result of a praxis based on critical reflection on reality (8).

However, what is observed in the practices of health services about Education and Communication is that they still need to adhere to models that are often inadequate to deal with the complexity of living with DM2. In general, health education actions are centered on prescriptive approaches based on transmitting information to achieve behavioral change and adherence to treatment. This approach cannot provide the patient with global knowledge of the disease, much less provide conditions for them to care for themselves. This type of education is called traditional health education because it involves activities focusing on diseases, how to avoid them, their implications, and their recovery, using prescriptive actions (9), covering biomedical topics related to the disease and its treatment.

Contemporary health education practices are dialogical, addressing people's daily lives, such as leisure, healthy eating, and popular knowledge (10). Furthermore, these interventions are person-centered and goal-setting to achieve health-related outcomes and improve quality of life (11).

Developing contemporary health education is expected to train people with self-management skills, that is, with knowledge, monitoring, management, and decision-making appropriate for health, to achieve a better quality of life and fewer complications. In this sense, health education programs must evaluate patients' behaviors and clinical and psychosocial aspects to adapt educational actions, consider different populations, and respect the race, belief, culture, education, and health knowledge of individuals, this can favor an increase of knowledge, skills, and motivation of people who have DM (12).

In an integrative literature review on the role of health education as a strategy for managing DM (7), the authors identified that the insertion of health education as a dialogical tool favors the construction of knowledge, constituting a facilitating strategy in managing DM. Therefore, health education is an effective method and provides several benefits for users with diabetes, mainly in constructing conscious self-care for metabolic control with quality of life.

In health education, it is expected to use methodological devices, which are applied to monitor chronic conditions and follow the specificities of each case, making the care plan natural and sustainable, significantly improving the quality of life of the people assisted. Using these contemporary technologies in managing the care of people with DM can contribute to achieving results, considering changes in behavior and lifestyle (13, 14).

From this perspective, this Research Topic aims to collect articles to expand our knowledge and understanding of appropriate strategies for health education with a focus on DM2. In this electronic collection, four articles cover the mentioned aspects. One study (Derakhshandeh-Rishehri et al.) evaluated the cost-effectiveness of a nutritional education program using group telecommunications vs. weblog (Web Tel), and the authors concluded that Web Tel was a more cost-effective method than the others tested and can be used as a priority in the education of people with DM.

A second study (Lamers-Johnsn et al.) on registered dietitians' care, aligned with the Academy of Nutrition and Dietetics' evidence-based nutritional practice guidelines for type 1 and 2 diabetes, identified that the proposed methodology is not as effective since the recommendations most frequently implemented by people with DM were related only to improving glycemic control, and we know that health education requires broader care than just glycemic control.

The third study (Chattopadhyay et al.) developed a clinical guideline for managing T2DM in adults by Ayurvedic professionals. In this study, it is observed that the guideline produced provides essential information about DM2, more from a traditional perspective, and it is not possible to infer whether the tool constructs knowledge and self-care for people with DM2.

The last article (Ali et al.) presents the creation of an innovative storytelling technique for better treatment of DM2. This resource seems to bring the daily life of people with DM closer to the everyday and be a resource for the practice of contemporary health education, as it is a method that can provide several benefits for users with diabetes, especially building conscious self-care for metabolic control and improves the quality of life.

## Author contributions

LN: Writing—original draft, Writing—review & editing, Conceptualization, Methodology. ES: Writing—original draft, Writing—review & editing, Conceptualization, Methodology.
